# Sex differences in diet-induced MASLD – are female mice naturally protected?

**DOI:** 10.3389/fendo.2025.1567573

**Published:** 2025-03-14

**Authors:** Jana Meyer, Ana Mendes Teixeira, Sandy Richter, Dean P. Larner, Asifuddin Syed, Nora Klöting, Madlen Matz-Soja, Susanne Gaul, Anja Barnikol-Oettler, Wieland Kiess, Diana Le Duc, Melanie Penke, Antje Garten

**Affiliations:** ^1^ Center for Pediatric Research, University Hospital for Children and Adolescents, Leipzig University, Leipzig, Germany; ^2^ Department of Biosciences, School of Science and Technology, Nottingham Trent University, Nottingham, United Kingdom; ^3^ Helmholtz Institute for Metabolic, Obesity and Vascular Research (HI-MAG) belonging to Helmholtz Center Munich at the University and University Hospital, Leipzig, Germany; ^4^ Division of Hepatology, Clinic and Polyclinic for Oncology, Gastroenterology, Hepatology, and Pneumology, University Hospital Leipzig, Leipzig, Germany; ^5^ Klinik und Poliklinik für Kardiologie, University Hospital Leipzig, Leipzig University, Leipzig, Germany; ^6^ Institute of Human Genetics, University Medical Center Leipzig, Leipzig, Germany; ^7^ Department of Evolutionary Genetics, Max Planck Institute for Evolutionary Anthropology, Leipzig, Germany

**Keywords:** MAFLD, NAFLD, high fat diet, adipose tissue, fibrosis, estrogen, collagen I

## Abstract

Males suffer more often from profibrotic changes in liver than females. The underlying mechanism for this sex difference in the prevalence and manifestation of Metabolic dysfunction-associated Steatotic Liver Disease (MASLD) is not yet completely known. We studied male and female mice that were induced to develop MASLD by consuming a “fast food” diet (FFD) and assessed metabolic phenotype as well as liver histology and compared them with mice fed with a matched control diet (CD). Our aim was to check for sex-specific differences in MASLD development in a mouse model of diet-induced profibrotic changes in the liver. Our results demonstrate a clear difference in body weight, fat distribution and changes in liver tissue for male and female mice fed with FFD. We found that female mice stored lipids mainly in subcutaneous and visceral adipose tissue while males increased ectopic lipid accumulation in the liver which resulted in hepatomegaly and increased *transforming growth factor β 1* (*Tgfb1*) and *collagen I* (*Col1a1*) expression concomitant to fibrosis development. This was absent in female mice. Analysis of estrogen receptor -α *(Esr1)* and -β *(Esr2)* expression revealed an upregulation of *Esr2* in livers of male FFD-fed mice whereas in female liver tissue a higher expression in *Esr1* could be observed. This study supports *Esr1* and *Esr2* as potential targets to reverse negative effects of diet-induced profibrotic changes in the liver.

## 1 Introduction

Metabolic dysfunction-associated Fatty Liver Disease (MAFLD) (1) or Metabolic dysfunction-associated Steatotic Liver Disease (MASLD) (2), previously named non-alcoholic fatty liver disease (NAFLD), is the major chronic liver disease worldwide (3).

MASLD denominates a spectrum of liver diseases ranging from simple accumulation of triglycerides in liver (steatosis) to inflammation (steatohepatitis, MASH) and fibrosis (4, 5). The disease is strongly correlated with obesity and insulin resistance (6).

Hepatic fibrosis is characterized by an excessive deposition of extracellular matrix (ECM) that could evolve to cirrhosis or hepatocellular carcinoma (7). Previously thought to be irreversible (8), a number of studies have shown a potential reversal of all stages of fibrosis (9, 10). For this reason, understanding the process of fibrogenesis allows the identification of markers of disease progression and offers a potential target for therapeutic intervention.

One possible target could be transforming growth factor beta (Tgf β), which is involved in all stages of MASLD progression. Tgf β plays a pivotal role in fibrosis development through inducing ECM protein production and activating hepatic stellate cells (HSC) (11). These liver injury activated HSCs have a key function in liver regeneration too, and are the key producers of collagen, the deposition of which is involved in the development of fibrosis and which is the most abundant component of ECM (6, 12, 13).

The incidence of MASLD is highest in obese children and adult men; however incidences also increase in menopausal and postmenopausal women (14–16). A groundbreaking study was published in 2000, supporting the notion that sexually dimorphic risk factors are associated with MASLD (17). Many studies suggest that estradiol (E_2_) can be responsible for these sex differences and variable incidence ratios. The estrogen receptors (Er) α and β are the mediators of estrogen action and expressed in adipose tissue. The precise role of Er α and β in MASLD development is not clarified. Previous studies demonstrated that hepatic steatosis occurred in *Esr1* knockout mice (18) but not in *Esr2* deficient male mice (19). In addition, estrogen deficiency promotes MASH progression in high-fat and high-cholesterol fed mice (20). Moreover, it has been shown that high fat diet-fed rats develop fatty liver and hepatic insulin resistance after three days of feeding (21). Fast food diet-fed mice show also higher levels of aspartate aminotransferase (ASAT) as indicator of hepatocyte damage compared to control diet fed mice (22).

Using this mouse model of diet-induced fibrosis MASH (22) we aimed to identify sex-specific differences in MASLD development and ascertain factors involved, which could be targeted to prevent fibrotic changes in metabolic liver disease.

## 2 Materials and methods

### 2.1 Chemicals and Reagents

Unless otherwise stated, chemicals were bought from Sigma-Aldrich (St Louis, USA).

### 2.2 Animal experiments

Mouse experiments were performed in accordance with the guidelines approved by the local authorities of the State of Saxony, Germany, as recommended by the responsible local animal ethics review board (Landesdirektion Saxony, Leipzig, TVV43/14). C57BL/6NCrl mice (28 male, 28 female), 6 weeks old, were purchased from the Medical Experimental Center, Leipzig University and randomized according to fat mass into 4 groups per sex (n=7 each). Mice were housed in groups of 3‐4 at 22 ± 2°C on a 12 h light/dark cycle with free access to feed and water, checked daily for signs of illness and weighed once a week.

Starting from an age of 8 weeks, mice of both sexes were fed either a control diet (CD88137, 5.1% crude fat, 23.2% sugar, no cholesterol) or the “fast food” diet, a modified Western diet (TD88137, Ssniff, Soest, Germany) containing 21.2% crude fat, 33.2% sugar and 2% cholesterol, providing 40% of energy as fat (milk fat, 12% saturated) for 16 or 24 weeks. This resulted in a total of 8 experimental groups, four per time point. Drinking water for both control and fast food diet groups was supplemented with 42 g/l sugar solution (55% fructose and 45% glucose). This dietary regimen has been described previously to recapitulate features of the metabolic syndrome and NASH with progressive fibrosis (22). Lean and fat mass were assessed by EchoMRI™ in week 8, 15 and 23. Intraperitoneal glucose tolerance tests (GTTs) were performed at the age of 16 and 24 weeks after an overnight fast of 12 h by injecting 2 g glucose per kg body weight. Blood samples for glucose measurements were taken from the tail vein after 0, 15, 30, 60, and 120 min and measured by using an automated glucose monitor (GlucoMen; Menarini Diagnostics, Wokingham, U.K.) as described previously (23). Mice were sacrificed by CO_2_ asphyxiation, followed by cardiac puncture for blood collection and by organ collection. Blood was incubated at room temperature for 1h and centrifuged at 10 min, 2500 x *g* for serum collection to measure liver enzymes and glycated haemoglobin (HbA1c). Organs (liver, subcutaneous fat (SAT), epididymal [visceral] fat (VAT)) were harvested, weighed and processed for histological and biochemical analyses or snap frozen in liquid nitrogen.

### 2.3 Laboratory analyses

HbA1c and activities of alanine aminotransferase (ALAT) and aspartate aminotransferase (ASAT) in serum were measured spectrophotometrically as indicators of hepatocellular disintegration and necrosis using a Cobas C111 analyzer (Roche Diagnostics; Rotkreuz, Switzerland) according to the manufacturer’s instructions.

### 2.4 Histological analyses

Adipose tissue histology, measurements of lipid droplet size and number and adipocyte size distributions analyses were performed as previously described (24). A liver lobe (*lobus hepatis sinister*) was fixed in 4% paraformaldehyde for 3 days, paraffin-embedded and stained with hematoxylin and eosin for histological evaluation of the percentage of liver fat. Hepatic steatosis was quantified using ImageJ (25)from 100x magnified TIFF micrographs (n = 5-7 images per experimental group) and represented as the percentage of vacuoles as a proxy for lipid accumulation present in each section. Picrosirius red staining was used for fibrillar collagen detection and quantified using ImageJ analysis of 200x magnified TIFF micrographs (26) (n=6-7 images per experimental group). Another lobe (*lobus medialis dexter*) was cryo-embedded in Tissue-Tek and cryo-sectioned (6 µm), fixed in 4% formalin and stained with Oil-Red O for lipid droplet quantification with ImageJ (n=4-6 images per experimental group) as described previously (27). For visualization, an EVOS FL Auto 2 microscope (Thermo Scientific) was used.

### 2.5 Gene expression analysis

Total RNA of liver tissue was extracted using TRIzol^®^ Reagent (Life Technologies) according to manufacturer’s protocol. 1 µg of total RNA was transcribed into cDNA by M-MLV Reverse Transcriptase (#28025013, Invitrogen). Quantitative PCR analyses were performed using the Absolute qPCR SYBR Green Low ROX Mix (Thermo Scientific) or qPCR Master Mix Plus ROX (Eurogentec) and the Applied Biosystems QuantStudio 3 System (Thermo Scientific). Gene expression values are shown as fold changes respective to male CD fed mice. *Cyclophilin b* alternatively designated as *peptidylprolyl isomerase b* (*Ppib*) or *hypoxanthin-phosphoribosyltransferase* (*Hprt*) were used as housekeeping genes for normalization. The specific primer sequences are listed in **Supplementary Table S1**.

### 2.6 Statistical analysis

All statistical analyses were performed using GraphPad Prism version 10.2.3 for Windows, GraphPad Software, Boston, Massachusetts USA, www.graphpad.com. Analyses comparing male and female mice on CD or FFD (sex and diet as independent variables) were performed using two-way analyses of variance (ANOVA) with subsequent Tukey´s multiple comparisons *post hoc* test. Differences in gene expression fold changes were tested with one sample *t*-test. All data were presented as means ± SD. Statistical significance was defined as p < 0.05.

## 3 Results

### 3.1 Female FFD-fed mice stored more fat in adipose tissue depots than males

**Figure 1:**
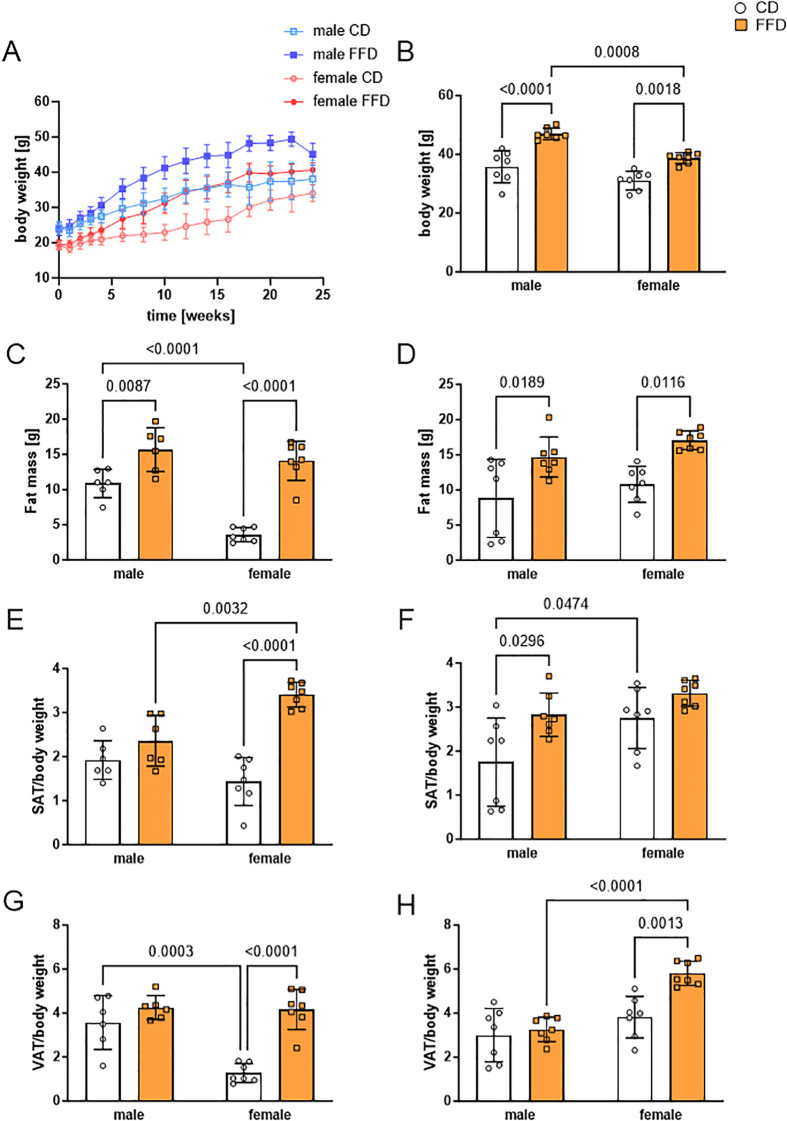
Fast food diet (FFD) fed mice showed sex-dependent differences in body weight, fat mass, and subcutaneous (SAT) or visceral (VAT) adipose tissue compared to control diet (CD) fed mice. **(A)** Body weight increase over the time course of the study (24 weeks). Male FFD mice in dark blue, male CD mice in light blue (both groups n = 13 until week 16, n = 7 until week 24), female FFD mice in dark red, female CD mice in light red (n = 14 until week 16, n = 7 until week 24). **(B)** At 24 weeks, male mice fed with FFD had a higher body weight compared to FFD-fed female (1.2fold, p=0.0008) and CD-fed male mice (1.3fold, p<0.0001). **(C)** At 16 weeks, fat mass of both FFD-fed groups was higher compared to CD-fed mice. CD-fed males had higher fat mass than CD-fed females (3fold, p<0.0001). **(D)** At 24 weeks, fat mass was increased in FFD-fed vs. CD-fed mice for both, males (1.7fold, p=0.0189) and females (1.6fold, p=0.0116). **(E)** At 16 weeks, SAT per body weight was higher in FFD female mice than in CD female mice (2.4fold, n=7 per group, p<0.0001) and FFD male mice (1.4fold, p=0.0032). **(F)** At 24 weeks, SAT per body weight was higher in FFD-fed compared to CD-fed male mice (1.6fold, p=0.0296). **(G)** At 16 weeks, VAT per body weight was higher in FFD-fed compared to CD-fed female mice (3.2fold, p<0.0001). Male CD-fed mice had higher VAT per body weight than female CD-fed mice (2.8fold, p=0.0003). **(H)** At 24 weeks, FFD-fed females had a higher VAT per body weight than both, CD-fed females (1.5fold, p=0.0013) and FFD-fed males (1.8fold, p<0.0001). Data are presented as mean ± SD, with points indicating 6-7 mice per group. Differences <0.05 were considered significant as determined by two-way analyses of variance (ANOVA) with subsequent Tukey´s multiple comparisons *post hoc* test.

To determine the sex-specific impact of FFD, we measured body weight, fat and lean mass, the weight of adipose tissue depots and mean adipocyte size after 16 and 24 weeks on the respective diets. As expected, body weight of all mice, regardless of sex and diet, increased over the course of time (**Figure 1A**). Male FFD-fed mice gained weight faster and had a higher mean weight at the end of the 24-week period (47.0g ± 2.0g, n=7) than FFD females (38.7g, ± 1.9g, n=7) and CD male mice (35.8g ± 5.5g, n=7). Female mice on CD had the lowest body weight (31.0g ± 3.2, n=7, **Figure 1B**).

We compared fat mass, subcutaneous adipose tissue (SAT) and visceral adipose tissue (VAT) normalized to body weight at 16 weeks with 24 weeks. Fat mass was higher in both male and female FFD-fed mice compared to CD-fed mice at both 16 and 24 weeks, while there were no differences in fat mass between males and females on FFD at either time point (**Figures 1C, D**). Interestingly, at 16 weeks, fat mass of CD-fed mice was higher in males (10.9g ± 2.0g, n=6) compared to females (3.6g ± 1.0g, n=7; p<0.0001, **Figure 1C**), which was not the case anymore at 24 weeks (males 15.7 ± 3.1g, females 14.1 ± 2.8g, **Figure 1D**). Lean mass was not different between diets at any time point, but higher in males than in females on their respective diet at 24 weeks (**Supplementary Figures S1A, B**).

To examine sex-specific differences in fat deposition, we took a closer look at SAT and VAT weight. At 16 weeks, female mice on FFD had significantly more SAT relative to their body weight (3.4 ± 0.3, n=7) than FFD-fed males (2.4 ± 0.6, n=6, p=0.00332, **Figure 1E**) and CD females (1.4 ± 0.5, n=7, p<0.0001; **Figure 1E**). At 24 weeks, the difference between CD and FFD-fed mice was significant only for males (1.8 ± 1.0 vs. 2.8 ± 0.5, n=7, p=0.0296), while CD-fed females had accumulated more SAT/body weight than CD-fed males (2.8 ± 0.7 vs. 1.8 ± 1.0, n=7, p=0.0474). There was no more difference in SAT/body weight between CD and FFD-fed females at 24 weeks (**Figure 1F**).

For VAT/body weight, there was neither a difference between FFD-fed males and females or between FFD and CD-fed males at 16 weeks (**Figure 1G**), while CD-fed females had significantly less VAT/body weight than FFD-fed females (1.3 ± 0.4 vs. 4.2 ± 0.9, n=7, p<0.0001, **Figure 1G**) or CD-fed males (1.3 ± 0.4 vs. 3.6 ± 1.2, n=7, p=0.0003, **Figure 1G**). At 24 weeks, FFD-fed females has increased VAT/body weight, so that it was significantly more compared to FFD-fed males (5.8 ± 0.6 vs. 3.3 ± 0.6, n=7, p<0.0001, **Figure 1H**).

Absolute SAT (**Supplementary Figures S1C, D**) and VAT (**Supplementary Figures S1E, F**) masses were similar to the normalized masses. No significant differences in adipocyte size could be measured after 16 (data not shown) or 24 weeks for both adipose tissue depots (**Supplementary Figures S1G, H**).

Glucose tolerance at 16 weeks was similar in FFD-fed mice and CD-fed males, with CD-fed females having a smaller area under the curve (AUC, **Supplementary Figures S2A, C**; p=0.0008 comparing CD fed male mice and p<0.0001 comparing female FFD mice, n=6-7) than the other experimental groups. This difference vanished at 24 weeks with AUCs being similar in all groups (**Supplementary Figures S2B, D**). Fasting blood glucose was higher in FFD-fed compared to CD-fed mice of the respective sex (**Supplementary Figures S2E, F**). HbA1c was higher in male compared to female mice, but not different between CD and FFD-fed mice (**Supplementary Figures S2G, H**).

Collectively, we found sex-specific differences of body weight and fat distribution between male and female mice. Female FFD-fed mice had more SAT then CD-fed females already at 16 weeks and increased their VAT depot at 24 weeks, whereas male mice on FFD had similar amounts of SAT than CD males and showed increased SAT at 24 weeks. This points to a sex-dependent difference in the preferred fat storage depot in this animal model.

### 3.2 Fast food diet causes lipid accumulation mainly in livers of male mice

**Figure 2:**
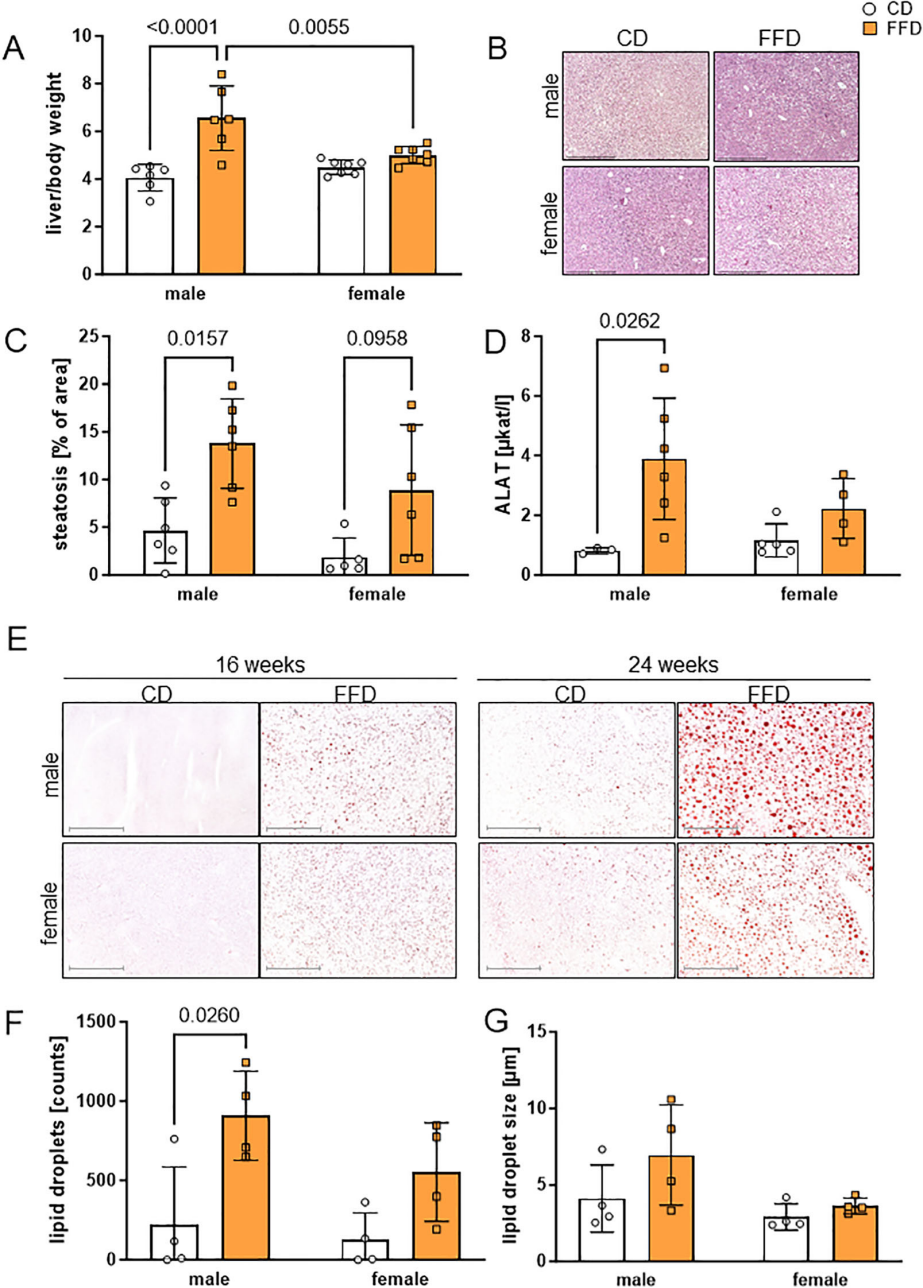
Fast food diet (FFD) was associated with progressive accumulation of lipids compared to control diet (CD) in male mouse livers. **(A)** At 16 weeks, weight of liver per body weight was higher for males on FFD compared to CD males (1.6fold, p<0.0001) and FFD females (1.3fold, p=0.0055). **(B)** Representative micrographs of male (upper) and female (lower) paraffin-embedded, hematoxylin/eosin stained mouse liver sections (100x magnification, scale bar 500 µm) at 16 weeks on CD (left) or FFD (right). **(C)** At 16 weeks, percentage of hepatic steatosis was higher in FFD males compared to CD males (3fold, p=0.0157) and showed a trend towards higher values in female livers (4.8fold, p=0.0958). **(D)** At 16 weeks, alanine aminotransferase (ALAT) was higher in male FFD compared to CD mice (4.7fold, p=0.0262). **(E)** Lipid droplet content in male and female mouse livers detected by Oil Red O staining at 16 and 24 weeks of CD or FFD feeding. Representative images for each time point, sex and diet are shown (magnification 100x, scale bar 500µm). Quantification of **(F)** lipid droplet number and **(G)** lipid droplet size after 16 weeks. Lipid droplet number was higher in livers from male FFD compared to CD mice (4.1fold, p=0.026). Data are presented as mean ± SD, points represent 3-7 mice per group, differences <0.05 were considered significant as determined by two-way analyses of variance (ANOVA) with subsequent Tukey´s multiple comparisons *post hoc* test.

We next checked for sex-specific differences regarding fat storage in the liver. Liver per body weight of 16 week old mice was significantly higher in FFD-fed compared to CD-fed males (1.6fold, n=6, p<0.0001) and compared to female FFD-fed mice (1.3fold, n=6 males, 7 females, p=0.0055, **Figure 2A**). Similar differences in liver/body weight were seen at 24 weeks in males (CD male 4.1± 0.8, n=7, FFD male 7.1 ± 1.3, n=7, p<0.0001). In 24 week old females, the liver weight difference between FFD and CD-fed mice became significant (CD female 4.4 ± 0.8, n=7, FFD female 6.0 ± 1.1, n=7, p=0.0247; **Supplementary Figure S3A**). The macroscopic differences between livers of male FFD and CD mice were also obvious, with livers from FFD mice being considerably bigger and paler than from CD mice (**Supplementary Figure S3B**). Similarly, absolute liver weights were also higher in FFD males (2.9g ± 0.7g, n=6) compared to CD males (1.5g ± 0.3, n=6, p<0.0001) and to FFD females (1.8g ± 0.3, n=7, p=0.0004) after 16 weeks (**Supplementary Figure S3C**) and after 24 weeks (**Supplementary Figure S3D**). This suggests that the amount of liver fat in females increased between 16 and 24 weeks. Moreover, livers from male FFD-fed mice were heavier than the FFD-fed female ones.

Liver lipid accumulation was visible as vacuoles in both male and female mouse livers from FFD-fed mice after 16 (**Figure 2B**) and 24 (**Supplementary Figure S4A**) weeks, but was more pronounced in livers from male FFD-fed mice. Quantification showed a significantly higher percentage of steatosis in FFD-fed compared to CD-fed mice at 16 weeks (males: 3fold, n=,6, p=0.0157; females 4.8fold, n=5,6, p=0.0958, **Figure 2C**). At 24 weeks, there was no significant difference between male and female mice on either diet (**Supplementary Figure S3E**).

Hepatocellular ballooning as a sign for hepatocyte damage could be observed in some livers from FFD-fed mice, both at 16 and 24 weeks (**Supplementary Figure S4B**). In contrast, we did not observe any signs of immune cell infiltration, based on images from H&E stained tissue (**Supplementary Figures S4A, B**).

In order to examine markers for liver damage, we measured serum liver enzymes alanine aminotransferase (ALAT) and aspartate aminotransferase (ASAT). After 16 weeks, ALAT was significantly higher in male FFD-fed compared to CD-fed mice (p=0.0262; **Figure 2D**), implying more damage to hepatocytes in FFD males. This difference persisted at 24 weeks (**Supplementary Figure S3F**). ASAT measurements did not show significant differences (data not shown).

To assess histological changes, more specifically the incorporation of lipids into hepatocytes, we measured lipid droplet content by Oil Red O (ORO) staining (**Figure 2E**). We found that livers from male mice after FFD feeding presented more and bigger lipid droplets than the female ones, especially at 24 weeks. Already at 16 weeks, males on FFD showed a 4.1fold higher number of hepatic lipid droplets compared to CD males (n=4, p=0.026, **Figure 2F**). Although there were more hepatic lipid droplets also in female mice on FFD compared to CD, the difference was not significant (**Figure 2F**). After 24 weeks, males on FFD showed an 8.5fold higher hepatic lipid droplet number than CD males (n=5, p=0.025), while in females, hepatic lipid droplet number was similar between CD and FFD-fed mice (**Supplementary Figure S3G**).

The size of hepatic lipid droplets was not significantly different between CD-fed and FFD-fed mice after 16 weeks (**Figure 2G**). After 24 weeks, FFD-fed mice of both sexes showed larger hepatic lipid droplets (males 5.4 ± 0.5 µm compared to 3.1 ± 0.5 µm, n=5; p=0.0004, females 4.9 ± 1.0 µm compared to 3.2 ± 0.5 µm, n=6, p=0.0023) compared to the respective CD-fed mice (**Supplementary Figure S3H**).

### 3.3 FFD feeding promotes signs of fibrosis only in male mouse livers

**Figure 3:**
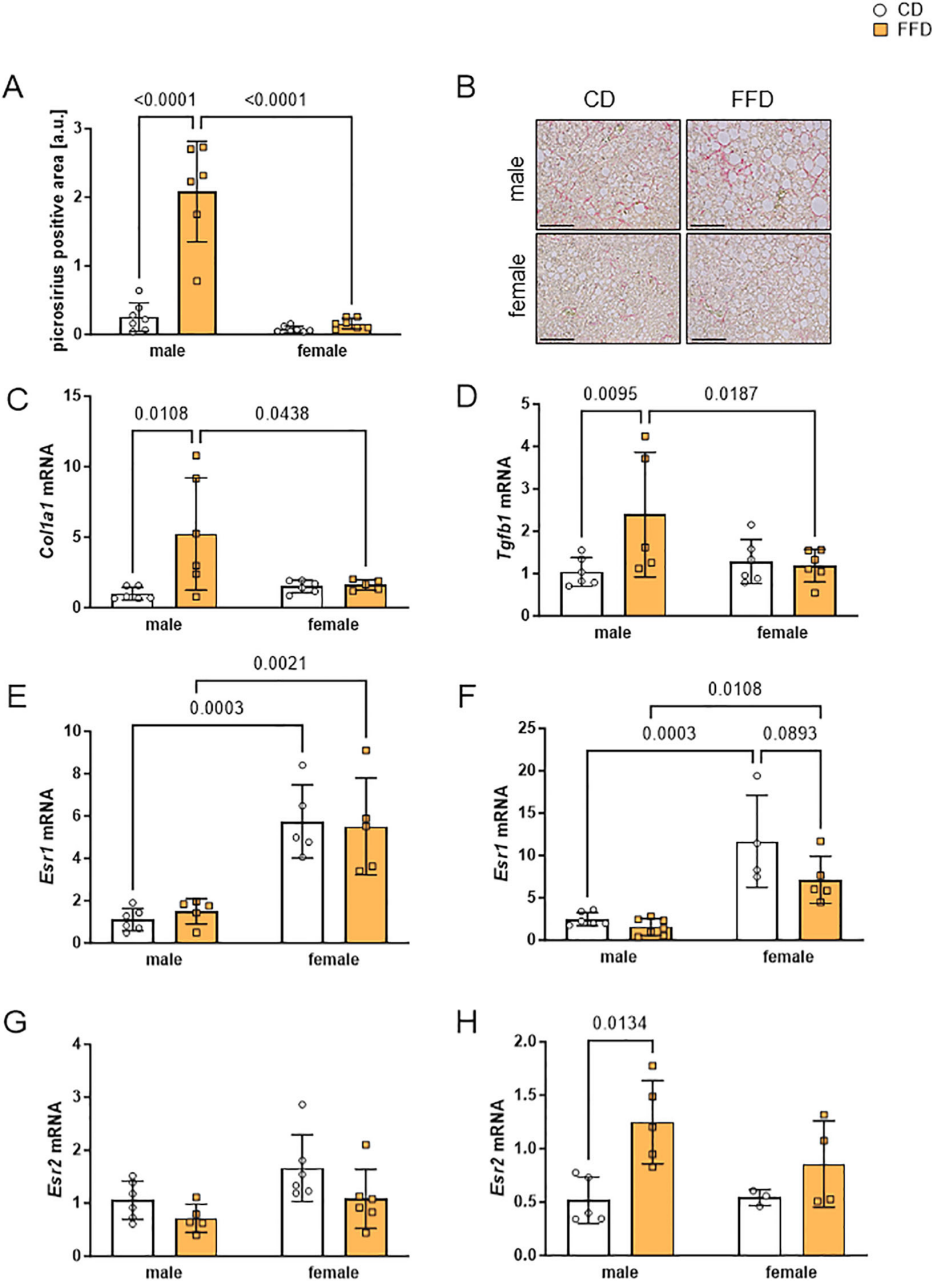
Livers from male fast food diet (FFD)-fed mice showed profibrotic changes and gene expression differences compared to FFD females and control diet (CD)-fed males. **(A)** Densitometric quantification of picrosirius red (PSR)-positive areas at 24 weeks showed more PSR-positive stained liver tissue in FFD males compared to CD males (8.1fold, p<0.0001) or to FFD females (13fold, p<0.0001). **(B)** Representative images of PSR- stained liver sections from male (upper) and female (lower) mice on CD (left) or FFD (right) for 24 weeks (magnification 200x, scale bar 200 µm). Hepatic gene expression was analyzed at 16 weeks on CD or FFD of **(C)** *collagen I* (*Col1a1)* and **(D)** *transforming growth factor beta 1* (*Tgfb1). Col1A1* and *Tgfb1* were increased in FFD males compared to CD males (*Col1A1*: 5.2fold, p=0.0108; *Tgfb1:*2.4fold, p=0.0095) and to FFD females (*Col1A1:* 3.2fold, p=0.0438; *Tgfb1:*2fold, p=0.0187). Gene expression of *estrogen receptor α* (*Esr1)* after **(E)** 16 and **(F)** 24 weeks. At 16 weeks, *Esr1* expression was 6fold higher in female mouse livers regardless of diet. At 24 weeks, *Esr1* expression in livers from female CD mice was 4.7fold (p=0.0003) and from female FFD mice 4.6fold (p=0.0108) increased compared to livers from male mice on the respective diet. Gene expression of *estrogen receptor β* (*Esr2)* after **(G)** 16 and **(H)** 24 weeks. Hepatic expression of *Esr2* was similar in all groups after 16 weeks. At 24 weeks, *Esr2* expression was higher in male FFD-fed compared to CD-fed mice (2.4fold, p=0.0134). Data are shown as mean fold changes ± SD related to expression values in CD male mice, with points indicating 4-7 mice per group. *Cyclophilin b (Ppib)* or *hypoxanthine phosphoribosyltransferase (Hprt)* were used as housekeeping genes. Statistical significance was defined as p<0.05 and tested by one sample *t*-test.

Given the increased amount of hepatic lipids and significantly higher ALAT serum levels in male FFD-fed mice, we next examined the extent of collagen deposition as a measure for profibrotic changes at 16 and 24 weeks. We did not see picrosirius red (PSR) staining after 16 weeks (data not shown), but found more PSR positive areas in livers of male FFD-fed mice compared to CD-fed mice after 24 weeks. Quantification of staining suggested a higher amount of collagen deposition in livers of FFD-fed male mice compared to either FFD-fed females or CD-fed mice, however with a large phenotypic variation (FFD males: 2.1 ± 0.7, n=6, vs. FFD females: 0.2 ± 0.1, p<0.0001, **Figure 3A**). There were no obvious PSR positive areas in livers from female mice on either diet or CD fed mice (**Figure 3B**). This finding was supported by significantly increased hepatic *collagen I* (*Col1a1*) expression in male FFD-fed mice, which was detected already after 16 weeks compared to CD-fed males (5.2fold, n=6, p=0.0108, **Figure 3C**) and FFD-fed females (3.2fold, n=5, p=0.0438; **Figure 3C**). The same was seen at 24 weeks with livers of FFD-fed males showing a 7-fold higher *Col1a1* expression compared to CD-fed males (n=5-6, p=0.0050, **Supplementary Figure S5A**) and 2.9fold higher compared to FFD-fed females (n=5 - 6, p=0.0365, **Supplementary Figure S5A**).

We then asked what factors could contribute to increased collagen synthesis and measured the mRNA expression of *transforming growth factor beta 1 (Tgfb1)*, which we hypothesized to be higher in livers of FFD-fed mice. In fact, at 16 weeks FFD feeding was associated with an increase in *Tgfb1* expression in male mouse livers compared to male CD-fed mice (2.4fold, n=5-6, p=0.0095) and to female FFD-fed mice (2fold, n=5-6, p=0.0187, **Figure 3D**). At 24 weeks, livers of male FFD-fed mice showed the highest *Tgfb1* expression (4fold higher in comparison to male CD, n=6, p=0.0315, **Supplementary Figure S4B**), but no significant difference to FFD-fed females (**Supplementary Figure S4B**). It is interesting to note that hepatic *Tgfb1* expression in female mice hardly changed, regardless of diet or time point. Gene expression of the gluconeogenic enzyme *phosphoenolpyruvate carboxykinase* (*Pepck*, **Supplementary Figure S5C**) and *glucose-6-phosphate dehydrogenase* (*G6pdh*, **Supplementary Figure S5D**), involved in the pentose phosphate pathway, was higher in livers of male FFD-fed compared to CD-fed male mice after 16 weeks, with a significant difference for *Pepck* expression (1.8fold, n=6, p=0.0128) and a trend towards significantly different *Pepck* expression compared to female FFD mice (**Supplementary Figure S5C**).

Because estradiol levels are obviously different between male and female mice and estradiol is known as a factor protecting from metabolic liver disease, we analyze *estrogen receptors alpha* (*Esr1*) and beta (*Esr2*) gene expression. *Esr1* and *Esr2* had different expression patterns (**Figures 3E-H**). At 16 weeks, female livers presented 4fold higher *Esr1* expression compared to male livers after CD (n=5-6, p=0.0003, **Figure 3E**) or FFD-feeding (n=5, p=0.0021, **Figure 3E**). At 24 weeks, hepatic *Esr1* expression in female mice was 4.7fold higher compared to males (CD, n=4-6, p=0.0003; FFD, n=5-6, p=0.0108; **Figure 3F**), but we saw a trend towards higher hepatic *Esr1* expression in female CD-fed mice compared to FFD-fed females (1.6fold higher in CD vs. FFD, n=4,5, p=0.0893). Hepatic *Esr2* expression was similar in all conditions at 16 weeks (**Figure 3G**). After 24 weeks, livers from male FFD mice demonstrated a higher *Esr2* expression compared to CD males (2.4fold, n=5, p=0.0134, **Figure 3H**).

## 4 Discussion

Both the prevalence and incidence of MASLD are increasing dramatically worldwide, reaching epidemic proportions with its onset occurring at younger ages in recent years (28–31). MASLD is now the second leading indication for liver transplantation (32) and accounts for an increasing proportion of hepatocellular carcinoma (33). The need to understand underlying pathomechanisms is therefore very considerable and medically significant.

According to the World Health Organization, the main reason for this dramatic trend in MASLD and Metabolic Dysfunction-associated Steatohepatitis (MASH) is the rising prevalence of obesity worldwide (34), which is partly due to increased food intake and a sedentary lifestyle (35). However, it should be noted that there are sex-specific differences. MASLD mainly and increasingly affects adult men (17, 36). Generally, women of fertile age have a lower risk of MASLD than men, while this protection is lost after menopause, when women have a MASLD prevalence comparable to men (14, 37). The underlying causes are still not fully understood. To learn more about this sexual dimorphism in diet-induced hepatic fibrosis development of MASLD we used a mouse model of metabolic liver disease induced by a “fast food diet” (FFD) high in saturated fat, fructose and cholesterol that was described to induce profibrotic changes and hepatocyte damage in murine liver (22). We compared the phenotype of male with that of female mice and found key differences in fat distribution, gene expression and fibrosis development. Of note, in the original study (22), both male and female mice were included in each experimental group without mentioning sex-dependent differences in phenotype.

As expected, both male and female mice on FFD gained more weight over the course of the study than mice on CD, with a bigger increase in male compared to female mice on FFD, as has previously been shown in other studies (14, 22, 38). Checking WAT depot mass at two time points during MASLD development, we could show that, in contrast to males, female mice on FFD primarily showed an increase in subcutaneous and, subsequently, visceral white adipose tissue (WAT) depots. Sex differences in fat deposition and mobilization in adipose tissue have previously been described in mice (39) and humans (40–42), with predominant lipid accumulation in subcutaneous WAT in females and visceral WAT in males. Interestingly, adipose tissue of the visceral WAT compartment contributes more to hepatic fatty acid uptake than subcutaneous WAT because of its anatomical position (43).

We also found sex-dependent differences in hepatic lipid accumulation, with male mice having bigger livers with a higher percentage of steatosis and more lipid droplets already after 16 weeks on FFD. While they did not quite reach the liver/body weight ratio reported by Charlton et al. (7.1 ± 1.3 vs. 8.5), male FFD-fed mice in our study also did not accumulate substantially more hepatic lipids at 24 (7.1 ± 1.3) than at 16 weeks (6.6 ± 1.4). In contrast, female FFD-fed mice reached a higher liver/body weight ratio than CD-fed only at 24 weeks, pointing to a delayed storage of lipids in the liver. This delay of adverse effects of FFD in females was also seen when checking serum levels of ALAT and number of lipid droplets. Large lipid droplets are a hallmark of steatosis (44, 45) There is evidence that the accumulation of lipids in the liver is linked to liver fibrosis, inflammation, apoptosis and cancer (11, 46). Blood glucose levels during GTT were strikingly lower in CD-fed female mice compared to the other groups at 16 weeks, pointing to a faster glucose metabolism and mirroring the lower overall fat mass as well as visceral fat per body weight in CD-fed females. This difference was lost completely at 24 weeks, when glucose tolerance was similar between all groups regardless of diet or sex. In contrast, Charlton et al. observed a significant higher blood glucose level after weeks on fast food vs. control diet or a high fat diet, indicating a bigger impact of fast food diet on glucose metabolism than in our study. Other differences included a higher level of ASAT and higher expression of myofibroblast activation marker anti-smooth muscle actin (ASMA, data not shown) (22), for both parameters we did not detect differences. There are several potential reasons for these differences in phenotype, such as different mouse strains (C57/B6J vs. C57/B6NCrl) and different animal facility (47), which was shown to exerts a mayor influence on mouse phenotypes (47, 48).

Tgf β has been recognized as a key molecular regulator in hepatic fibrosis. Its fibrogenic effects are mainly associated with hepatic stellate cell (HSC) activation and the production of extracellular matrix (ECM) protein, e.g. collagen (11). Moreover, Tgf β signaling in hepatocytes under metabolic stress mediates hepatocyte death and lipid accumulation (49, 50), which are processes leading to the development of steatohepatitis (50).

Our study revealed that in contrast to fat accumulation, *Tgfb1* and *Col1a1* expression increased over time in male livers, suggesting that fibrosis was exacerbated with prolonged FFD feeding. This points to a sex difference in fibrosis development, as we did not observe these gene expression changes in the female mouse livers.

Previous studies have shown that estradiol prevented reactive oxygen species and *Tgfb1* production in cultured rat HSC (51). Interestingly, another study showed that the fibrogenic genes, *Tgfb1* and *Col1a1*, were upregulated in the livers of female ovariectomized and high-fat/high-cholesterol-fed mice (20). Estradiol and its derivatives were previously shown to be potent endogenous antioxidants that reduce lipid peroxide levels, are linked to fat metabolism in the liver (52, 53) and associated with sex-specific differences in fibrosis development (14, 36, 54).

Because hepatic estrogen receptors (Er) α and/or β mediate estrogen action (54), we asked whether the expression of these receptors shows sex-dependent differences in mouse livers, which could provide an explanation for the previously observed sex-specific differences in fibrosis development. A recent rodent study revealed that Er α, but not Er β, plays an essential role together with peroxisome proliferator-activated receptor-γ coactivator 1 α (Pgc1a). Expression of Pgc1a was shown to be inversely correlated with liver fat and MASLD severity. Er α partnering with Pgc1a is associated with a reduction of oxidative stress damage and impairs the transition from steatosis to severe steatohepatitis (55). Furthermore, *Esr1* knockout mice develop hepatic steatosis more often than their wild-type controls as a consequence of the increased expression of genes involved in *de novo* lipogenesis (18, 56). Similar protective effects of Er α were also found in other studies (57–59). In contrast to these studies, hepatic Er α was shown to be not required for the protection against FFD-induced hepatic steatosis in female mice and did not mediate sexual dimorphism in liver mitochondria function (60). Er β expression was found to be favorable in different animal models of liver injury (61, 62) mainly acting through suppression of hepatic stellate cell activation (63).

In our study, *Esr1* and *Esr2* mRNA levels encoding for Er α and β, respectively, were similar in livers from 16-week-old male mice, but differed in female livers, with a higher expression of *Esr1* in female mouse livers at 16 weeks, which was, however, not dependent on diet. High hepatic *Esr1* expression was also reported in other studies, where normal rat livers primarily express *Esr1* with low levels of *Esr2* (64). Interestingly, in our study, expression of *Esr2* was doubled in male livers from FFD-fed mice after 24 weeks, while there was a trend towards higher *Esr2* expression, but no significant changes in *Esr1* expression in female livers. This could suggest a compensatory upregulation of hepatic *Esr2* in males to prevent further damage after prolonged FFD.

In humans, the occurrence of MASLD was found to be higher in menopausal and postmenopausal than in premenopausal women (36). Besides, ovarian senescence, via hypo-estrogenemia, facilitates both the development of massive hepatic steatosis and the fibrotic progression of liver disease (65). The creation of novel medications based on the protective effects of estrogens could provide new therapeutic strategies for the treatment of MASLD (66). Our results support this proposed concept and additionally point specifically to Er β as potential target for novel MASLD treatment options.

In summary, we found that a diet high in fat, fructose and cholesterol led to an increased fat accumulation and an upregulation of profibrotic factors in livers of male mice, while female mice appeared to store excess fat mainly in subcutaneous and visceral adipose tissue depots. We also saw a diet- and time-associated change in expression patterns of estrogen receptors which was more pronounced in male mouse livers. Female mice seemed to be protected against these profibrotic changes.

## Data Availability

The raw data supporting the conclusions of this article will be made available by the authors, without undue reservation.
