# Supplementary material for: Sex differences in diet-induced MASLD – are female mice naturally protected?
Source: Front Endocrinol (Lausanne). 2025 Mar 14;16:1567573. doi: 10.3389/fendo.2025.1567573 (PMC11949793; doi:10.3389/fendo.2025.1567573)
Supplement: Supplementary file 1 [file DataSheet1.docx]

Suppl. Figure S1

Suppl. Figure S2

**
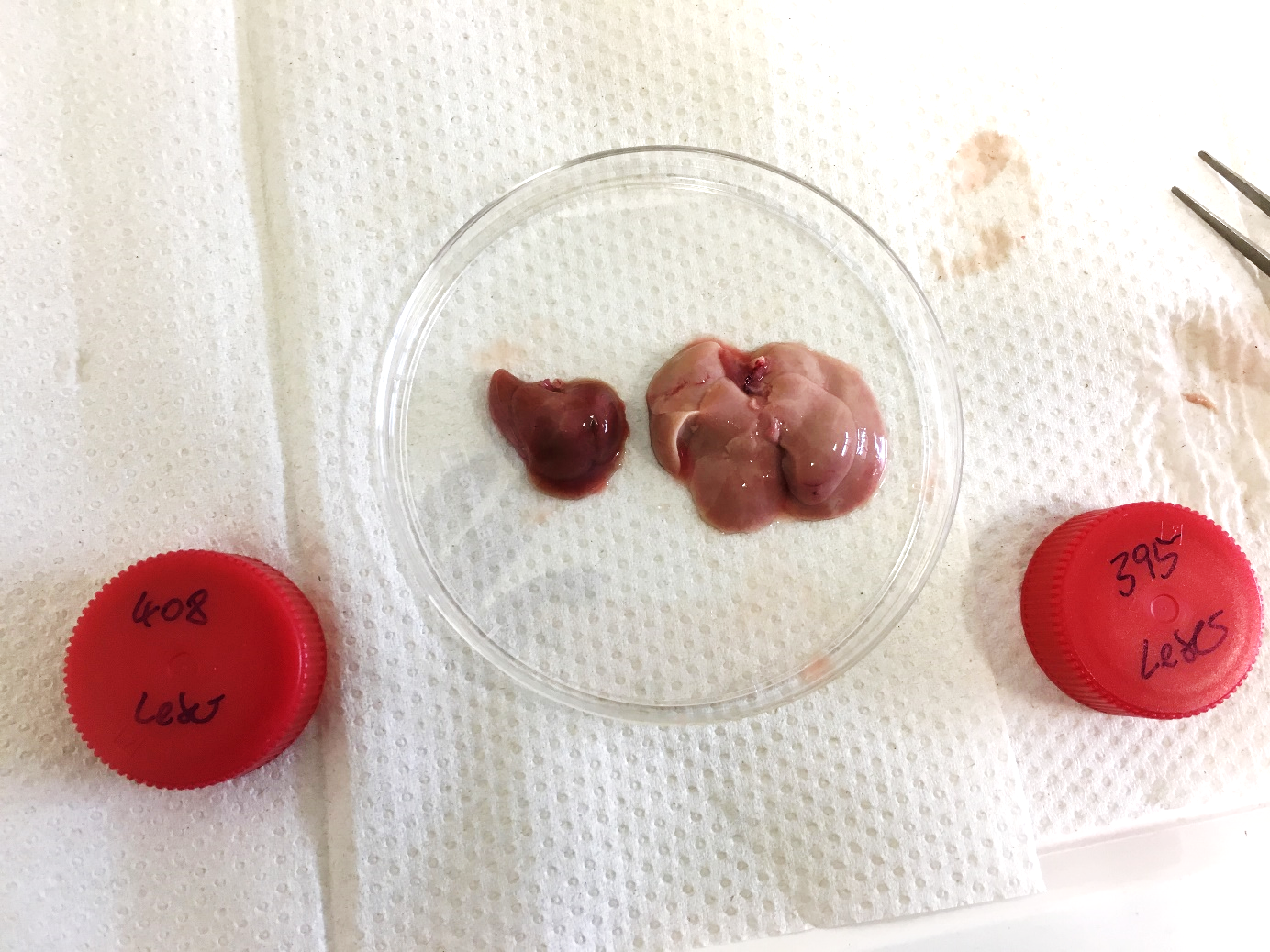
**

Suppl. Figure S3

male

female

Suppl. Figure S4

**
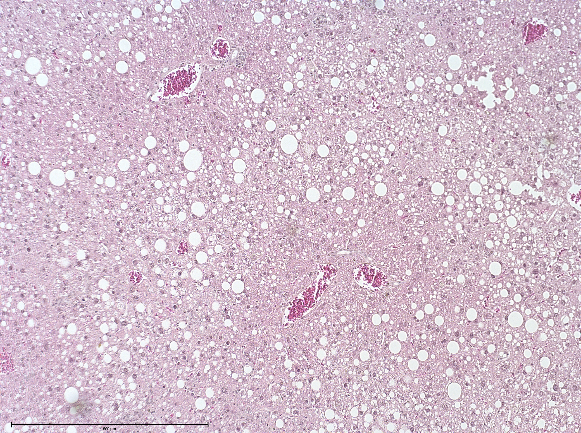

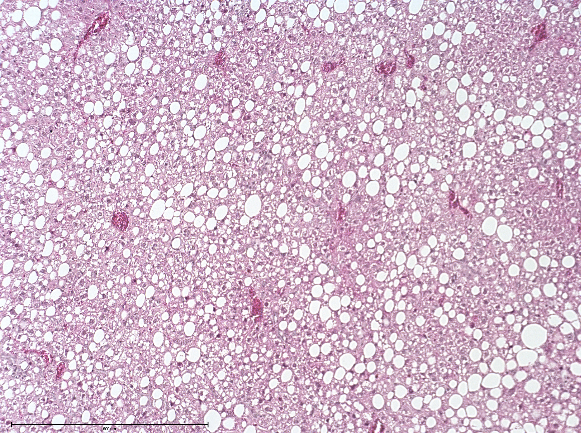

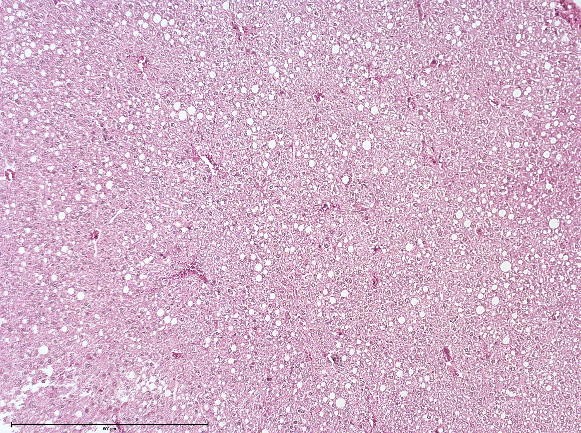

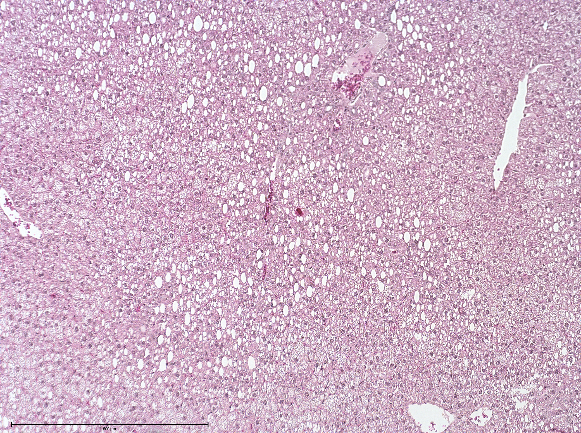
**

A


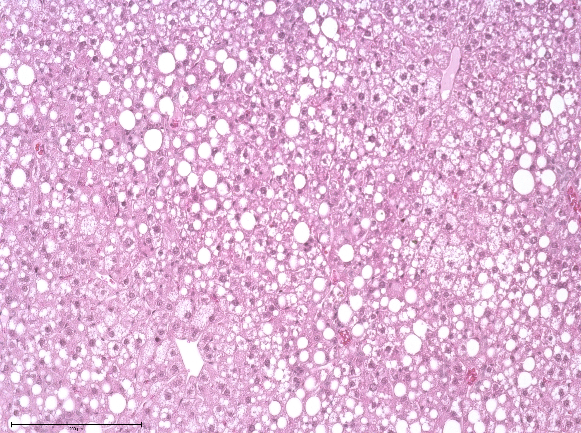

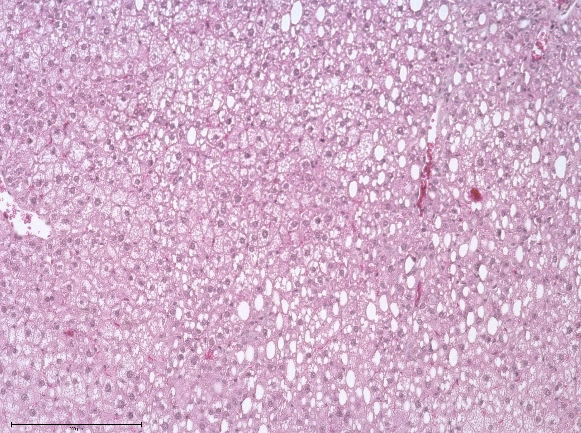

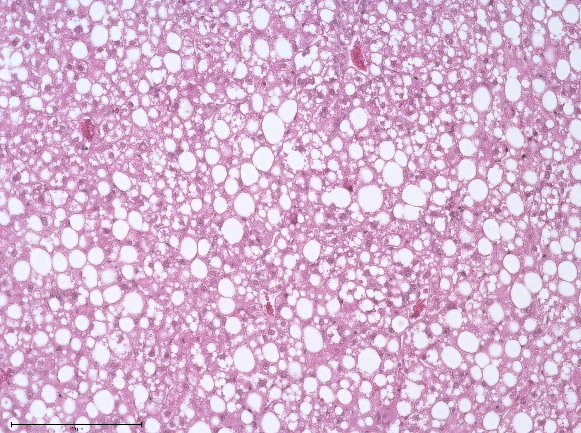

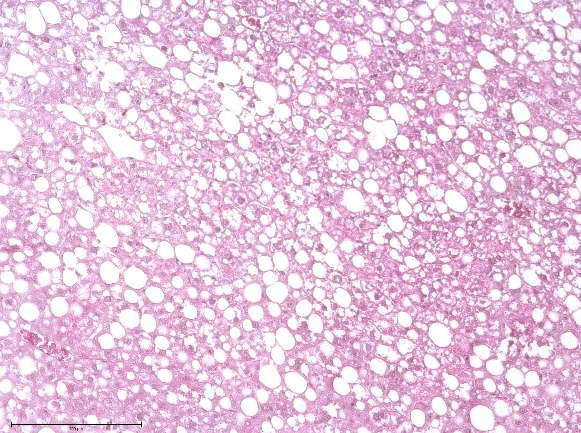


24 wk

CD

24 wk

FFD

16 wk

FFD

24 wk

FFD

female

male

B

Suppl. Figure S5

**Supplementary Figure S1. Fast Food Diet (FFD)-fed mice show sex-dependent differences in fat and lean mass and adipose tissue depots compared to Control Diet (CD)-fed mice.**

Lean mass of mice after **A)** 16 weeks and **B)** 24 weeks was not different on either diet. Absolute subcutaneous adipose tissue (SAT) mass was higher in FFD-fed compared to CD-fed mice after **C)** 16 and **D)** 24 weeks. After 16 weeks, CD-fed female mice had lower SAT mass than CD-fed males. Absolute visceral adipose tissue (VAT) mass was higher in FFD-fed compared to CD-fed mice after **E)** 16 and **F)** 24 weeks. After 16 weeks, CD-fed female mice had lower VAT mass than CD-fed males. FFD-fed females had higher VAT mass than FFD-fed males after 24 weeks. No differences were seen in mean adipocyte size in **G)** SAT or **H)** VAT after 24 weeks. Data are presented as mean ± SD, points represent 6-7 mice per group. Statistical significance was defined as p<0.05 and tested by 2 way analysis of variance (ANOVA) for each time point followed by Tukey´s multiple comparisons post hoc test.

**Supplementary Figure S2. Glucose tolerance and HbA1c are not different in fast food diet (FFD) compared to control diet (CD) fed mice at 24 weeks.**

Time course of intraperitoneal glucose tolerance tests (GTTs) at **A)** 16 and **B)** 24 weeks. GTTs were performed after an overnight fast of 12 h by injecting 2 g glucose per kg body weight. Blood samples for glucose measurements were taken after 0, 15, 30, 60, and 120 min. **C)** At 16 weeks, female CD-fed mice had lower area under the curve than both female FFD-fed mice (0.7fold, p<0.0001) and male CD-fed mice (0.7fold, p=0.0008). **D)** There was no difference at 24 weeks. Fasting blood glucose at **E)** 16 (1.7fold, p<0.001 for males and 2fold, n=7, p<0.0001 for females) and **F)** 24 weeks (1.9fold, n=7, p<0.001 for males and 2fold, n=7, p<0.0001 for females) was higher in FFD compared to CD-fed mice of the respective sex, while HbA1c at **G)** 16 (1.1fold, p=0.0046 for CD-fed and 1.1fold, p=0.078) and **H)** 24 weeks (1.1fold, p=0.0213 for CD-fed and 1.2fold, p=0.013) was slightly higher in male than in female mice regardless of diet. Data are presented as mean ± SD, points represent 5-7 mice per group. Statistical significance was defined as p<0.05 and tested by 2 way analysis of variance (ANOVA) for each time point followed by Tukey´s multiple comparisons post hoc test.

**Supplementary Figure S3. Fast food diet (FFD) causes progressive enlargement and fat accumulation in male mouse livers.**

**A)** Liver per body weight at 16 weeks was higher in fast food diet (FFD)-fed compared to control diet (CD)-fed mice of the respective sex. **B)** Appearance of livers from male (CD, left) or FFD (right)-fed mice at 24 weeks. Absolute liver weight at **C)** 16 weeks and **D)** 24 weeks was higher in FFD-fed males compared to FFD-fed females and in FFD-fed mice compared to CD-fed mice of the respective sex. **E)** Hepatic steatosis (percentage of area covered by vacuoles) was quantified by ImageJ analysis of hematoxylin & eosin-stained and paraffin embedded mouse liver sections (100x magnification). **F)** Alanine aminotransferase (ALAT) at 16 weeks was higher in serum of FFD males than CD males. **G)** After 24 weeks, FFD males showed a 8.5fold higher number of lipid droplets than the CD males, lipid droplet number in female livers was not different. **H)** Both male and female FFD-fed mice had bigger lipid droplets than CD-fed mice of the respective sex after 24 weeks as determined by Oil Red O staining of frozen liver sections (100x magnification). All data are presented as mean ± SD, points represent 6-7 mice per group, except for F) ALAT measurement (3-7 mice). Statistical significance was defined as p<0.05 and tested by 2 way analysis of variance (ANOVA) for each time point followed by Tukey´s multiple comparisons post hoc test.

.

**Supplementary Fig S4. Hematoxylin & eosin (H&E) stained liver sections show pronounced hepatic lipid accumulation in male mice on fast food diet (FFD).**

**A)** Paraffin-embedded, (H&E) stained liver sections (6 µm thickness, magnification 100x, scale bar = 500 µm) from 24 week old male (left), and female (right) mice on control diet (CD, upper panel), or fast food diet (FFD, lower panel) show a higher number of vacuoles in male FFD-fed mice. One representative micrograph per group is shown.

**B)** A) Paraffin-embedded, (H&E) stained liver sections (6 µm thickness, magnification 200x, scale bar = 200 µm) from 16 week old (upper panel), and 24 week old (lower panel), male (left), and female (right) mice on fast food diet (FFD) show vacuoles representing lipid accumulation indicated by black chevrons and hepatocyte ballooning indicated by black arrows. One representative micrograph per group is shown.

**Supplementary Fig S5. Sex-dependent hepatic gene expression changes of fast food diet (FFD) compared to control diet (CD)-fed mice.**

At 24 weeks, **A)** *Collagen I (Col1a1)* mRNA levels in livers from male mice fed with FFD were 7fold higher than in CD male livers and 3fold higher than in female FFD-fed mice**. B)** Hepatic *transforming growth factor beta* (*Tgfb1)* mRNA expression was higher in FFD-fed compared to CD-fed males at 24 weeks. At 16 weeks, gene expression of **C)** *phosphoenolpyruvate carboxykinase* (*Pepck*) was higher in FFD-fed compared to CD-fed males and trended to be higher than in FFD-fed females, while expression of **D)** glucose-6-phosphate dehydrogenase (*G6pdh*) was not significantly different.

Data are presented as mean±SD, points represent 5-6 mice per group. Gene expression values are shown as fold changes respective to male CD fed mice. *Cyclophilin B* (*Ppdib*) or *hypoxanthine phosphoribosyltransferase* (*Hprt*) were used as housekeeping genes. Statistical significance was defined as p<0.05 and tested by one sample *t*-test.
