# Supplementary material for: Sex differences in diet-induced MASLD – are female mice naturally protected?
Source: Front Endocrinol (Lausanne). 2025 Mar 14;16:1567573. doi: 10.3389/fendo.2025.1567573 (PMC11949793; doi:10.3389/fendo.2025.1567573)
Supplement: Supplementary file 2 [file Table1.docx]

**Supplementary Table S1. Primer and probe sequences for quantitative PCR analyses**

| **Gene name** | **Symbol** | **Forward primer** | **Reverse primer** | **Probe** |
| --- | --- | --- | --- | --- |
| Transforming growth factor beta | *Tgfb1* | AAG TTG GCA TGG TAG CCC TT | GCC CTG GAT ACCAAC TAT TGC | n.a. |
| Collagen 1A1 | *Col1A1* | CCC CGA GGC TCT GAA GGT C | GGA GCA CCA TTG GCA CCT TT | n.a. |
| Estrogen receptor alpha | *Esr1* | Mm00433149_m1, Thermo | | |
| Estrogen receptor beta | *Esr2* | Mm00599821_m1, Thermo | | |
| Phosphoenolpyruvate carboxykinase | *Pepck* | GTG CAT GAA AGG CCG CACCA | GAT CCG CAT GCT GGC CAC C | n.a. |
| Glucose-6-phosphate dehydrogenase | *G6pdh* | CCT GTG AGA CCG GAC CAG | AAA GAT AGC AAG AGT AGA AGT GAC CAT | n.a. |
| Cyclophilin | *Ppdib* | ATG TGG TTT TCG GCA AAG TT | TGA CAT CCT TCA GTG GCT TG | n.a. |
| Hypoxanthine phosphoribosyltransferase | *Hprt* | GGC AGT ATA ATC CAA AGA TGG TCA A | GTC TGG CTT ATA TCC AAC ACT TCG T | CAA GCT TGC TGG TGA AAA GGA CCC C |
